# Redetermined structure of oxaline: absolute configuration using Cu *K*α radiation

**DOI:** 10.1107/S1600536812019423

**Published:** 2012-05-05

**Authors:** Peng Qu, Zhi-Yong Wu, Wei-Ming Zhu

**Affiliations:** a Key Laboratory of Marine Drugs of the Ministry of Education of China, School of Medicine and Pharmacy, Ocean University of China, 266003 Qingdao, People’s Republic of China

## Abstract

In the title compound, C_24_H_25_N_5_O_4_, the stereogenic C atom bonded to three N atoms and one C atom has an *S* configuration and its directly bonded neighbour has an *R* configuration. An intra­molecular N—H⋯O hydrogen bond supports the near coplanarity of the two C_3_N_2_-five-membered rings [dihedral angle = 5.64 (10)°]. In the crystal, mol­ecules are linked by N—H⋯N hydrogen bonds, forming a *C*(8) chain propagating in [001]. The chains are connected by C—H⋯O inter­actions, generating a three-dimensional network. The previous study [Nagel *et al.* (1974 ▶). *Chem. Commun.* pp. 1021–1022] did not establish the absolute structure and no atomic coordinates were published or deposited.

## Related literature

For the previous structure, see: Nagel *et al.* (1974 ▶). For background to oxaline and its properties, see: Steyn (1970 ▶); Koizumi *et al.* (2004 ▶). For puckering parameters, see: Cremer & Pople (1975 ▶).
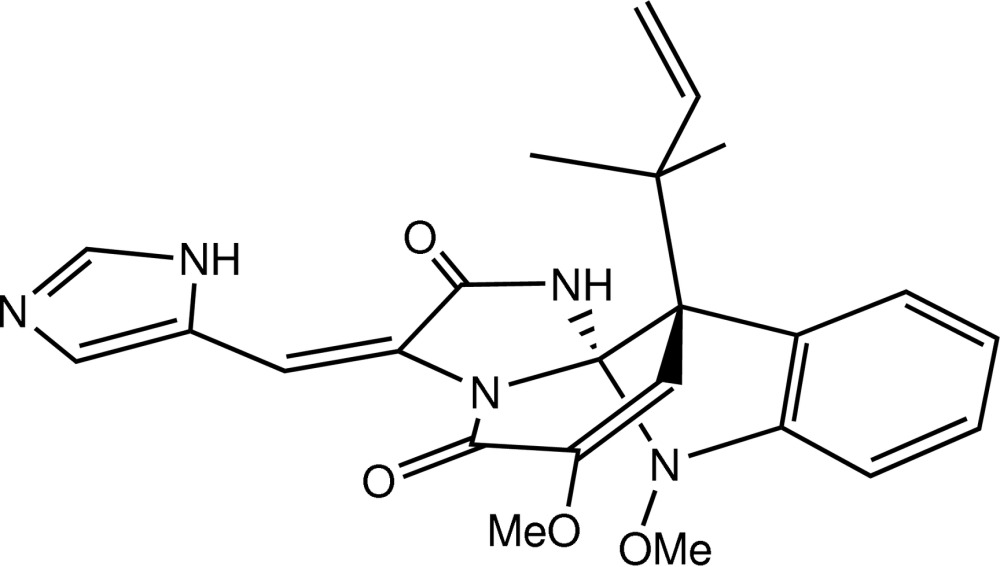


## Experimental

### 

#### Crystal data

C_24_H_25_N_5_O_4_
*M* *_r_* = 447.49
Orthorhombic, 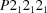

*a* = 10.7897 (2) Å
*b* = 13.2457 (3) Å
*c* = 15.6436 (4) Å
*V* = 2235.74 (9) Å^3^
*Z* = 4
Cu *K*α radiation
μ = 0.76 mm^−1^
*T* = 100 K
0.60 × 0.20 × 0.12 mm

#### Data collection

Bruker APEXII CCD diffractometer
Absorption correction: multi-scan (*SADABS*; Sheldrick, 2003 ▶) *T*_min_ = 0.658, *T*_max_ = 0.914
11202 measured reflections
3786 independent reflections
3766 reflections with *I* > 2σ(*I*)
*R*_int_ = 0.034

#### Refinement

*R*[*F* ^2^ > 2σ(*F* ^2^)] = 0.035
*wR*(*F* ^2^) = 0.089
*S* = 1.08
3786 reflections
379 parameters
H atoms treated by a mixture of independent and constrained refinement
Δρ_max_ = 0.64 e Å^−3^
Δρ_min_ = −0.26 e Å^−3^
Absolute structure: Flack (1983 ▶), 1403 Friedel pairs
Flack parameter: −0.05 (18)

### 

Data collection: *APEX2* (Bruker, 2004 ▶); cell refinement: *SAINT* (Bruker, 2004 ▶); data reduction: *SAINT*; program(s) used to solve structure: *SHELXS97* (Sheldrick, 2008 ▶); program(s) used to refine structure: *SHELXL97* (Sheldrick, 2008 ▶); molecular graphics: *XP* in *SHELXTL* (Sheldrick, 2008 ▶); software used to prepare material for publication: *WinGX* (Farrugia, 1999 ▶).

## Supplementary Material

Crystal structure: contains datablock(s) I, global. DOI: 10.1107/S1600536812019423/hb6738sup1.cif

Structure factors: contains datablock(s) I. DOI: 10.1107/S1600536812019423/hb6738Isup2.hkl

Additional supplementary materials:  crystallographic information; 3D view; checkCIF report

## Figures and Tables

**Table 1:** Hydrogen-bond geometry (Å, °)

*D*—H⋯*A*	*D*—H	H⋯*A*	*D*⋯*A*	*D*—H⋯*A*
N17—H17⋯O13	0.89 (2)	1.85 (2)	2.6562 (19)	151 (2)
N14—H14⋯N19^i^	0.83 (2)	1.97 (2)	2.798 (2)	175 (2)
C4—H4⋯O9^ii^	0.97 (2)	2.54 (2)	3.154 (2)	121.5 (16)
C20—H20⋯O13^iii^	1.00 (2)	2.54 (2)	3.353 (2)	137.9 (16)
C24—H24*C*⋯O13^iv^	0.98 (3)	2.47 (3)	3.382 (2)	154 (2)

Symmetry codes: (i) 
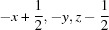
; (ii) 
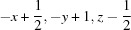
; (iii) 
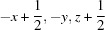
; (iv) 
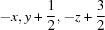
.

**Fig. 1.**
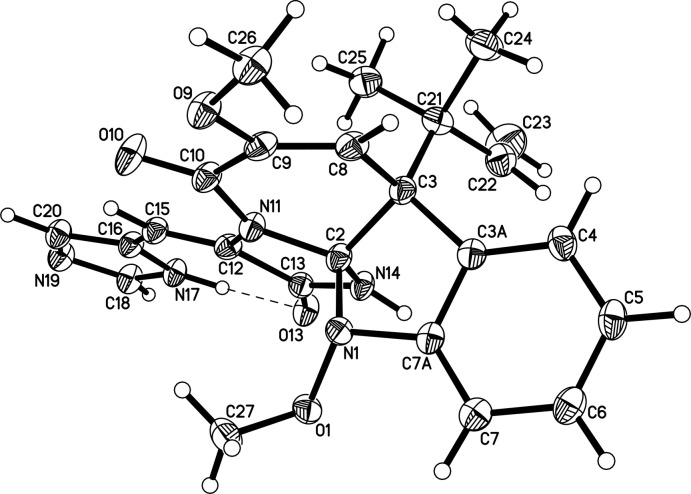
The molecular structure of (I), with displacement ellipsoids shown at the 50% probability level. Dashed lines indicates a intramolecular hydrogen bond.

**Fig. 2.**
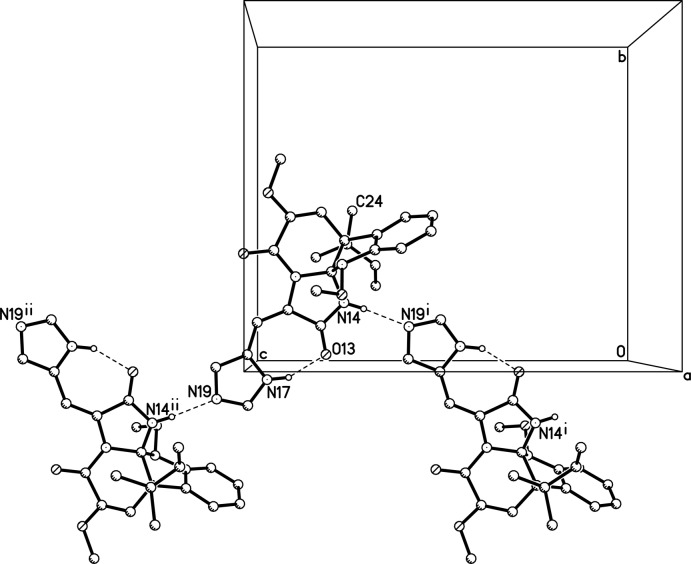
A one-dimensional classical hydrogen-bonding chain along the c axis. [Symmetry code: (i) 1/2 - x, -y, z - 1/2; (ii) 1/2 - x, -y, z + 1/2]

**Fig. 3.**
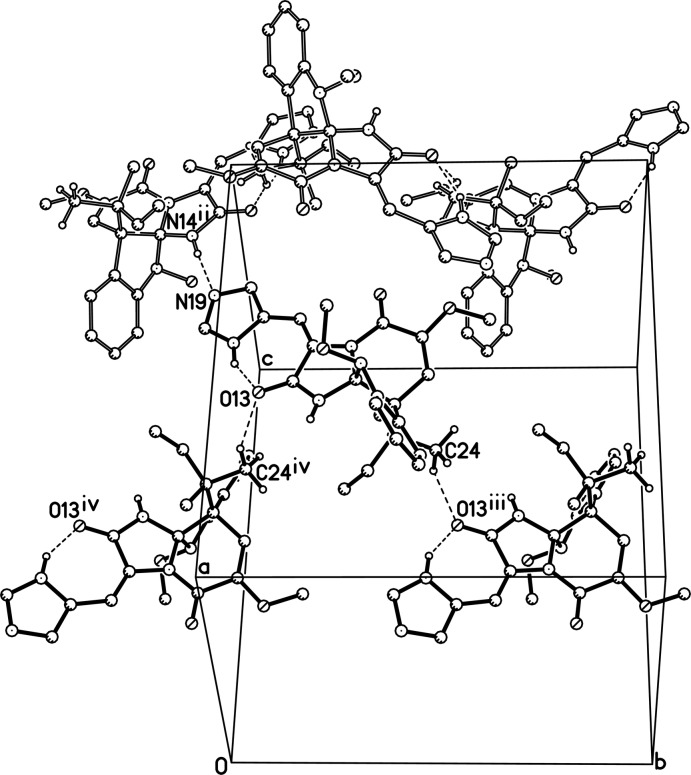
A view of a three-dimensional hydrogen-bonding networks assembled by the classical chains above and the nonclassical ones parallel to the b axis. [Symmetry code: (ii) 1/2 - x, -y, z + 1/2; (iii) -x, y + 1/2, 3/2 - z; (iv) -x, y - 1/2, 3/2 - z]

## supplementary crystallographic information

### Comment

Oxaline is a member of a class of biologically active indole alkaloids, characterized by a unique indoline spiroaminal framework and substitution of a 1,1-dimethylallyl ("reverse-prenyl") group at the benzylic ring junction. Oxaline was originally isolated from the culture broth of *Penicillium oxalicum* HK14–01 containing several unique structural features, including the N-OMe group, the unusual coupling of tryptophan and histidine, a single carbon atom bearing three nitrogen functionalities and a reversed prenyl group (Steyn, 1970). Besides, oxaline was found to inhibit tubulin polymerization in Jarkat cells, resulting in cell cycle arrest at the *M* phase (Koizumi *et al.*, 2004). The X-ray structure of oxaline on Mo—Kα data was determined without definite absolute configuration (Nagel, *et al.*, 1974). We isolated oxaline as part of our ongoing studies on characterizing bioactive metabolites from marine-derived halotolerant fungi. And the crystal structure on Cu—Kα and the absolute configuration are reported here.

The title compound I contains a four fused rings structure as illustrated in Fig. 1. Two chiral atoms of C2 and C3 have the absolute configurations of *S* and *R*, respectively. Atom of N1 is *S* but it can invert in solution. Atom O1 in I (*S-*) has a short intra-contact of O1···N14 [2.7018 (19) Å]. While in *R-* one, the short contact are 2.882(C3), 2.581(C8), 2.390(C9), 2.662(C10) and 2.675 Å(N11), which indicates a unfavorable configuration. Both bonds of C8═C9 and C12═C15 are *E* but *cis* conformation. The five-membered ring of N1—C2—C3—C3A—C7A adopts envelope conformation with the puckering parameters (Cremer and Pople, 1975) of Q[0.3968 (17) Å] and φ[34.8 (2)°]. The six-membered ring of C2—C3—C8—C9—C10—N11 has the puckering parameters of Q = 0.4342 (17) Å, θ = 69.0 (2)° and φ = 76.8 (2)°, which implies a conformation among boat, twist-boat and half-chair.

In the crystal, there are a one-dimensional classical hydrogen bonding chain parallel to the *c* axis (Fig. 2, Table 2) and a non-classical one along the *b* axis. These two kinds of chains together weave a three-dimensional supramolecular structure (Fig. 3).

### Experimental

The halotolerant fugal strain *Penicillium chrysogenum* HK14–01, was isolated from the sediments collected in the Yellow River Delta, Dongying, Shandong, China. The working strain was cultured under static conditions at 298 K for 35 days in two hundred 1*L* conical flasks containing the liquid medium (300 ml/flask) composed of glucose (10 g/*L*), peptone (5 g/*L*), yeast extract (3 g/*L*), malt extract (1.5 g/*L*), marinum salt (100 g/*L*). The fermented whole broth (60 *L*) was filtered through cheese cloth to separate into supernatant and mycelia. The mycelia was extracted three times with acetone. The acetone solution was concentrated under reduced pressure to afford an aqueous solution. The acetone solution was extracted three times with ethyl acetate to give an ethyl acetate solution which was concentrated under reduced pressure to give a crude extract (39 g). The crude extract, which was subjected to chromatography over silica gel column using a stepwise gradient elution of CH_2_Cl_2_/petroleum ether(50–100%,*V*/*V*) and CH_2_Cl_2_/MeOH (0–100%,*V*/*V*),to yield twelve fractions (Fr.1-Fr.12). Fr.9, was fractionated on a C-18 ODS column using a step gradient elution of MeOH/H_2_O (60–100%,*V*/*V*) and was separated into 6 subfractions (Fr.9.1-Fr.9.6). Fr.9.3 was applied on Sephadex LH-20 using CH_2_Cl_2_/MeOH (1:1) to yield the title compound (145.0 mg). Colourless blocks were obtained by slow evaporation of petroleum ether/acetone (1:1) solution at 298 K.

### Refinement

H atoms on C23 and C25 were placed in calculated positions, with C—H distances of 0.95 (C23) and 0.98 Å (C25), and were included in the final cycles of refinement in a riding model, with *U*_iso_(H) values equal to 1.2*U*_eq_(C23) or 1.5*U*_eq_(C25). All other H atoms were located in a difference Fourier map and included in structure-factor calculations with free refinement. The highest difference peak is 0.83Å from atom H25C.

### Crystal data

**Table:**

C_24_H_25_N_5_O_4_	*F*(000) = 944
*M**_r_* = 447.49	*D*_x_ = 1.329 Mg m^−^^3^
Orthorhombic, *P*2_1_2_1_2_1_	Cu *K*α radiation, λ = 1.54178 Å
Hall symbol: P 2ac 2ab	Cell parameters from 9936 reflections
*a* = 10.7897 (2) Å	θ = 2.8–69.0°
*b* = 13.2457 (3) Å	µ = 0.76 mm^−^^1^
*c* = 15.6436 (4) Å	*T* = 100 K
*V* = 2235.74 (9) Å^3^	Block, colourless
*Z* = 4	0.60 × 0.20 × 0.12 mm

### Data collection

**Table:**

Bruker APEXII CCD diffractometer	3786 independent reflections
Radiation source: fine-focus sealed tube	3766 reflections with *I* > 2σ(*I*)
Graphite monochromator	*R*_int_ = 0.034
φ and ω scans	θ_max_ = 69.4°, θ_min_ = 4.4°
Absorption correction: multi-scan (*SADABS*; Sheldrick, 2003)	*h* = −13→12
*T*_min_ = 0.658, *T*_max_ = 0.914	*k* = −15→15
11202 measured reflections	*l* = −16→18

### Refinement

**Table:**

Refinement on *F*^2^	Secondary atom site location: difference Fourier map
Least-squares matrix: full	Hydrogen site location: inferred from neighbouring sites
*R*[*F*^2^ > 2σ(*F*^2^)] = 0.035	H atoms treated by a mixture of independent and constrained refinement
*wR*(*F*^2^) = 0.089	*w* = 1/[σ^2^(*F*_o_^2^) + (0.0539*P*)^2^ + 0.608*P*] where *P* = (*F*_o_^2^ + 2*F*_c_^2^)/3
*S* = 1.08	(Δ/σ)_max_ < 0.001
3786 reflections	Δρ_max_ = 0.64 e Å^−^^3^
379 parameters	Δρ_min_ = −0.26 e Å^−^^3^
0 restraints	Absolute structure: Flack (1983), 1403 Friedel pairs
Primary atom site location: structure-invariant direct methods	Flack parameter: −0.05 (18)

### Special details

**Table:**

Geometry. All s.u.'s (except the s.u. in the dihedral angle between two l.s. planes) are estimated using the full covariance matrix. The cell s.u.'s are taken into account individually in the estimation of s.u.'s in distances, angles and torsion angles; correlations between s.u.'s in cell parameters are only used when they are defined by crystal symmetry. An approximate (isotropic) treatment of cell s.u.'s is used for estimating s.u.'s involving l.s. planes.
Refinement. Refinement of *F*^2^ against ALL reflections. The weighted *R*-factor *wR* and goodness of fit *S* are based on *F*^2^, conventional *R*-factors *R* are based on *F*, with *F* set to zero for negative *F*^2^. The threshold expression of *F*^2^ > 2σ(*F*^2^) is used only for calculating *R*-factors(gt) *etc*. and is not relevant to the choice of reflections for refinement. *R*-factors based on *F*^2^ are statistically about twice as large as those based on *F*, and *R*- factors based on ALL data will be even larger.

### Fractional atomic coordinates and isotropic or equivalent isotropic displacement parameters (Å^2^)

**Table:**

	*x*	*y*	*z*	*U*_iso_*/*U*_eq_	
C2	0.32699 (15)	0.27923 (12)	0.80027 (10)	0.0186 (3)	
C3	0.26044 (15)	0.37581 (12)	0.76650 (10)	0.0195 (3)	
C3A	0.33279 (15)	0.39156 (12)	0.68341 (11)	0.0194 (3)	
C4	0.30763 (17)	0.44534 (13)	0.60975 (11)	0.0229 (4)	
C5	0.39516 (18)	0.44605 (14)	0.54394 (11)	0.0253 (4)	
C6	0.50732 (17)	0.39574 (14)	0.55266 (12)	0.0246 (4)	
C7	0.53572 (16)	0.34468 (13)	0.62767 (11)	0.0220 (4)	
C7A	0.44724 (15)	0.34255 (12)	0.69104 (10)	0.0182 (3)	
C8	0.29907 (16)	0.46075 (12)	0.82645 (11)	0.0211 (4)	
C9	0.34100 (16)	0.44460 (12)	0.90560 (11)	0.0210 (3)	
C10	0.33551 (16)	0.34357 (13)	0.94788 (11)	0.0214 (3)	
C12	0.28689 (15)	0.16359 (13)	0.91257 (11)	0.0190 (3)	
C13	0.26417 (14)	0.11484 (12)	0.82818 (10)	0.0187 (3)	
C15	0.27783 (15)	0.12521 (13)	0.99230 (11)	0.0200 (3)	
C16	0.24075 (15)	0.02576 (13)	1.01914 (10)	0.0204 (3)	
C18	0.18204 (17)	−0.13354 (13)	1.01993 (11)	0.0233 (4)	
C20	0.24315 (17)	−0.01288 (13)	1.10109 (11)	0.0231 (3)	
C21	0.11422 (16)	0.36829 (14)	0.75912 (12)	0.0245 (4)	
C22	0.07620 (17)	0.30327 (15)	0.68323 (13)	0.0292 (4)	
C23	−0.0179 (2)	0.23927 (19)	0.68053 (16)	0.0467 (6)	
H23A	−0.0694	0.2306	0.7293	0.056*	
H23B	−0.0341	0.2020	0.6299	0.056*	
C24	0.06073 (19)	0.47547 (16)	0.74467 (14)	0.0319 (4)	
C25	0.05692 (16)	0.32850 (15)	0.84208 (12)	0.0281 (4)	
H25A	−0.0330	0.3387	0.8407	0.042*	
H25B	0.0921	0.3650	0.8908	0.042*	
H25C	0.0750	0.2563	0.8478	0.042*	
C26	0.38241 (19)	0.61629 (14)	0.92987 (12)	0.0261 (4)	
C27	0.62470 (18)	0.21991 (16)	0.83731 (13)	0.0304 (4)	
N1	0.45699 (13)	0.29884 (10)	0.77360 (9)	0.0185 (3)	
N11	0.32034 (13)	0.26396 (10)	0.89240 (9)	0.0194 (3)	
N14	0.28079 (13)	0.18525 (10)	0.76808 (9)	0.0192 (3)	
N17	0.19945 (13)	−0.05261 (11)	0.96885 (9)	0.0211 (3)	
N19	0.20655 (14)	−0.11236 (11)	1.10046 (9)	0.0243 (3)	
O1	0.52932 (11)	0.20898 (8)	0.77451 (8)	0.0220 (3)	
O9	0.38366 (12)	0.51505 (9)	0.96104 (8)	0.0242 (3)	
O10	0.33924 (13)	0.33375 (10)	1.02538 (8)	0.0297 (3)	
O13	0.23426 (11)	0.02613 (9)	0.81455 (7)	0.0227 (3)	
H4	0.230 (2)	0.4816 (18)	0.6029 (14)	0.036 (6)*	
H5	0.3779 (19)	0.4836 (16)	0.4900 (13)	0.023 (5)*	
H6	0.5638 (17)	0.3927 (14)	0.5057 (12)	0.015 (4)*	
H7	0.613 (2)	0.3112 (16)	0.6374 (13)	0.026 (5)*	
H8	0.2979 (17)	0.5235 (15)	0.8010 (12)	0.015 (4)*	
H14	0.284 (2)	0.1668 (17)	0.7172 (15)	0.027 (5)*	
H15	0.3009 (18)	0.1647 (16)	1.0401 (13)	0.021 (5)*	
H17	0.198 (2)	−0.0459 (17)	0.9125 (15)	0.029 (5)*	
H18	0.1494 (17)	−0.1977 (14)	0.9977 (12)	0.016 (4)*	
H20	0.269 (2)	0.0184 (16)	1.1564 (14)	0.028 (5)*	
H22	0.118 (3)	0.318 (2)	0.6324 (17)	0.050 (7)*	
H24A	0.095 (2)	0.5062 (18)	0.6938 (15)	0.037 (6)*	
H24B	0.080 (2)	0.522 (2)	0.7904 (16)	0.042 (7)*	
H24C	−0.028 (3)	0.4672 (19)	0.7331 (16)	0.045 (7)*	
H26A	0.4374 (17)	0.6236 (14)	0.8794 (12)	0.015 (4)*	
H26B	0.412 (2)	0.6584 (18)	0.9782 (16)	0.040 (6)*	
H26C	0.294 (2)	0.6392 (17)	0.9142 (14)	0.029 (5)*	
H27A	0.676 (2)	0.2766 (19)	0.8265 (15)	0.035 (6)*	
H27B	0.668 (2)	0.1612 (18)	0.8349 (15)	0.033 (6)*	
H27C	0.590 (2)	0.2315 (17)	0.8929 (14)	0.028 (5)*	

### Atomic displacement parameters (Å^2^)

**Table:**

	*U*^11^	*U*^22^	*U*^33^	*U*^12^	*U*^13^	*U*^23^
C2	0.0247 (8)	0.0158 (7)	0.0153 (8)	−0.0008 (6)	−0.0003 (6)	−0.0016 (6)
C3	0.0253 (8)	0.0165 (7)	0.0166 (8)	0.0022 (6)	0.0003 (6)	−0.0005 (6)
C3A	0.0255 (8)	0.0138 (7)	0.0189 (8)	−0.0011 (7)	−0.0012 (7)	−0.0018 (6)
C4	0.0291 (9)	0.0183 (8)	0.0213 (8)	0.0007 (7)	−0.0043 (7)	0.0012 (7)
C5	0.0352 (9)	0.0207 (9)	0.0199 (8)	−0.0039 (7)	−0.0023 (7)	0.0046 (7)
C6	0.0305 (9)	0.0223 (9)	0.0210 (8)	−0.0057 (7)	0.0028 (7)	0.0005 (7)
C7	0.0258 (8)	0.0180 (8)	0.0223 (8)	−0.0022 (7)	0.0005 (7)	−0.0026 (7)
C7A	0.0259 (8)	0.0120 (7)	0.0167 (7)	−0.0028 (6)	−0.0016 (6)	−0.0026 (6)
C8	0.0273 (8)	0.0132 (8)	0.0229 (9)	0.0008 (6)	0.0041 (7)	−0.0003 (7)
C9	0.0258 (8)	0.0165 (8)	0.0206 (8)	−0.0009 (7)	0.0045 (7)	−0.0040 (7)
C10	0.0286 (8)	0.0174 (8)	0.0181 (8)	−0.0010 (7)	0.0014 (7)	−0.0034 (6)
C12	0.0215 (7)	0.0157 (7)	0.0198 (8)	0.0002 (6)	0.0017 (6)	−0.0014 (7)
C13	0.0209 (7)	0.0170 (8)	0.0181 (8)	0.0009 (6)	−0.0005 (6)	0.0001 (6)
C15	0.0252 (8)	0.0173 (8)	0.0176 (8)	0.0007 (7)	0.0001 (6)	−0.0023 (6)
C16	0.0241 (8)	0.0191 (8)	0.0179 (8)	−0.0003 (7)	0.0006 (6)	−0.0021 (7)
C18	0.0310 (8)	0.0176 (8)	0.0213 (8)	−0.0049 (7)	0.0007 (7)	0.0020 (7)
C20	0.0306 (8)	0.0199 (8)	0.0190 (8)	−0.0018 (7)	0.0005 (7)	0.0003 (7)
C21	0.0238 (8)	0.0233 (9)	0.0264 (9)	0.0050 (7)	−0.0002 (7)	−0.0004 (7)
C22	0.0275 (9)	0.0345 (10)	0.0257 (9)	0.0009 (8)	−0.0031 (8)	−0.0011 (8)
C23	0.0451 (12)	0.0505 (13)	0.0446 (13)	−0.0061 (11)	0.0018 (11)	−0.0054 (11)
C24	0.0290 (10)	0.0293 (10)	0.0374 (11)	0.0085 (8)	0.0022 (8)	0.0036 (10)
C25	0.0258 (8)	0.0300 (9)	0.0286 (9)	0.0006 (7)	0.0018 (7)	0.0002 (8)
C26	0.0373 (10)	0.0160 (9)	0.0250 (9)	−0.0044 (8)	0.0029 (8)	−0.0023 (7)
C27	0.0309 (9)	0.0292 (10)	0.0310 (11)	0.0080 (8)	−0.0100 (8)	−0.0010 (8)
N1	0.0244 (7)	0.0121 (6)	0.0189 (7)	0.0027 (5)	0.0004 (6)	0.0024 (5)
N11	0.0274 (7)	0.0149 (7)	0.0161 (7)	−0.0006 (5)	0.0004 (5)	−0.0001 (5)
N14	0.0280 (7)	0.0152 (7)	0.0145 (7)	−0.0004 (5)	−0.0012 (5)	−0.0016 (5)
N17	0.0273 (7)	0.0195 (7)	0.0164 (7)	−0.0026 (6)	0.0004 (6)	0.0009 (6)
N19	0.0329 (7)	0.0188 (7)	0.0211 (7)	−0.0022 (6)	0.0019 (6)	0.0027 (6)
O1	0.0284 (6)	0.0152 (6)	0.0222 (6)	0.0062 (5)	−0.0023 (5)	−0.0006 (5)
O9	0.0369 (6)	0.0156 (6)	0.0202 (6)	−0.0040 (5)	0.0016 (5)	−0.0021 (5)
O10	0.0526 (8)	0.0206 (6)	0.0159 (6)	−0.0076 (6)	0.0019 (6)	−0.0022 (5)
O13	0.0340 (6)	0.0157 (6)	0.0184 (6)	−0.0052 (5)	−0.0009 (5)	−0.0011 (5)

### Geometric parameters (Å, º)

**Table:**

O1—N1	1.4233 (17)	C12—C13	1.490 (2)
O1—C27	1.430 (2)	C12—C15	1.350 (2)
O9—C9	1.354 (2)	C15—C16	1.439 (2)
O9—C26	1.427 (2)	C16—C20	1.381 (2)
O10—C10	1.220 (2)	C21—C22	1.523 (3)
O13—C13	1.237 (2)	C21—C24	1.549 (3)
N1—C2	1.486 (2)	C21—C25	1.531 (2)
N1—C7A	1.419 (2)	C22—C23	1.324 (3)
N11—C2	1.457 (2)	C4—H4	0.97 (3)
N11—C10	1.375 (2)	C5—H5	1.00 (2)
N11—C12	1.413 (2)	C6—H6	0.955 (19)
N14—C2	1.432 (2)	C7—H7	0.95 (2)
N14—C13	1.336 (2)	C8—H8	0.92 (2)
N17—C16	1.377 (2)	C15—H15	0.95 (2)
N17—C18	1.350 (2)	C18—H18	0.984 (19)
N19—C18	1.317 (2)	C20—H20	1.00 (2)
N19—C20	1.376 (2)	C22—H22	0.93 (3)
N14—H14	0.83 (2)	C23—H23A	0.9500
N17—H17	0.89 (2)	C23—H23B	0.9500
C2—C3	1.559 (2)	C24—H24A	0.97 (2)
C3—C3A	1.531 (2)	C24—H24B	0.97 (3)
C3—C8	1.523 (2)	C24—H24C	0.98 (3)
C3—C21	1.585 (2)	C25—H25A	0.9800
C3A—C4	1.382 (2)	C25—H25B	0.9800
C3A—C7A	1.400 (2)	C25—H25C	0.9800
C4—C5	1.397 (3)	C26—H26A	0.993 (19)
C5—C6	1.388 (3)	C26—H26B	0.99 (2)
C6—C7	1.389 (3)	C26—H26C	1.03 (2)
C7—C7A	1.377 (2)	C27—H27A	0.95 (2)
C8—C9	1.336 (2)	C27—H27B	0.91 (2)
C9—C10	1.494 (2)	C27—H27C	0.96 (2)
			
N1—O1—C27	108.47 (12)	N11—C12—C13	104.59 (13)
C9—O9—C26	115.18 (13)	N11—C12—C15	125.37 (16)
O1—N1—C2	111.62 (12)	C13—C12—C15	130.04 (15)
O1—N1—C7A	113.01 (12)	O13—C13—N14	125.21 (15)
C2—N1—C7A	104.88 (12)	O13—C13—C12	127.39 (15)
C2—N11—C10	120.79 (14)	N14—C13—C12	107.39 (13)
C2—N11—C12	111.34 (13)	C12—C15—C16	129.35 (16)
C10—N11—C12	127.64 (14)	C12—C15—H15	120.2 (13)
C2—N14—C13	113.94 (14)	C16—C15—H15	110.4 (13)
C2—N14—H14	125.3 (16)	N17—C16—C15	127.83 (15)
C13—N14—H14	118.2 (16)	N17—C16—C20	104.91 (14)
C16—N17—C18	107.78 (14)	C15—C16—C20	127.23 (16)
C16—N17—H17	120.0 (15)	N17—C18—N19	111.65 (15)
C18—N17—H17	131.7 (15)	N17—C18—H18	121.8 (11)
C18—N19—C20	105.57 (15)	N19—C18—H18	126.4 (11)
N1—C2—N11	110.39 (13)	N19—C20—C16	110.06 (15)
N1—C2—N14	112.43 (13)	N19—C20—H20	118.9 (12)
N1—C2—C3	101.31 (12)	C16—C20—H20	131.0 (12)
N11—C2—N14	102.12 (13)	C3—C21—C22	111.13 (14)
N11—C2—C3	115.23 (13)	C3—C21—C25	111.21 (14)
N14—C2—C3	115.70 (13)	C3—C21—C24	108.89 (15)
C2—C3—C3A	99.48 (13)	C22—C21—C24	107.71 (15)
C2—C3—C8	105.75 (13)	C22—C21—C25	110.94 (15)
C2—C3—C21	115.56 (14)	C24—C21—C25	106.77 (15)
C3A—C3—C8	106.43 (13)	C21—C22—C23	126.4 (2)
C3A—C3—C21	117.03 (14)	C21—C22—H22	114.8 (17)
C8—C3—C21	111.33 (14)	C23—C22—H22	118.2 (17)
C3—C3A—C4	132.71 (16)	C22—C23—H23A	120.0
C3—C3A—C7A	108.31 (14)	C22—C23—H23B	120.0
C4—C3A—C7A	118.92 (16)	H23A—C23—H23B	120.0
C3A—C4—C5	119.03 (17)	C21—C24—H24A	111.4 (14)
C3A—C4—H4	121.0 (14)	C21—C24—H24B	113.5 (15)
C5—C4—H4	119.9 (14)	H24A—C24—H24B	104.9 (19)
C4—C5—C6	120.91 (16)	C21—C24—H24C	106.7 (15)
C4—C5—H5	120.0 (12)	H24A—C24—H24C	105 (2)
C6—C5—H5	119.0 (12)	H24B—C24—H24C	115 (2)
C5—C6—C7	120.61 (17)	C21—C25—H25A	109.5
C5—C6—H6	120.1 (11)	C21—C25—H25B	109.5
C7—C6—H6	119.2 (11)	C21—C25—H25C	109.5
C6—C7—C7A	117.74 (16)	H25A—C25—H25B	109.5
C6—C7—H7	123.6 (13)	H25A—C25—H25C	109.5
C7A—C7—H7	118.6 (13)	H25B—C25—H25C	109.5
N1—C7A—C3A	109.40 (14)	O9—C26—H26A	111.0 (11)
N1—C7A—C7	127.75 (15)	O9—C26—H26B	105.4 (14)
C3A—C7A—C7	122.73 (15)	O9—C26—H26C	111.6 (13)
C3—C8—C9	123.06 (15)	H26A—C26—H26B	111.1 (18)
C3—C8—H8	113.4 (11)	H26A—C26—H26C	109.5 (16)
C9—C8—H8	123.3 (12)	H26B—C26—H26C	108.2 (19)
O9—C9—C8	126.76 (16)	O1—C27—H27A	112.0 (14)
O9—C9—C10	110.32 (14)	O1—C27—H27B	104.8 (14)
C8—C9—C10	122.71 (15)	O1—C27—H27C	111.2 (13)
O10—C10—N11	123.31 (16)	H27A—C27—H27B	112.0 (19)
O10—C10—C9	122.29 (15)	H27A—C27—H27C	105.0 (19)
N11—C10—C9	114.34 (14)	H27B—C27—H27C	112.0 (19)
			
C2—N1—O1—C27	−116.05 (15)	C12—C15—C16—N17	−3.6 (3)
C7A—N1—O1—C27	126.02 (15)	C12—C15—C16—C20	173.90 (17)
C8—C9—O9—C26	0.1 (2)	N17—C16—C20—N19	1.06 (19)
C10—C9—O9—C26	−174.67 (15)	C15—C16—C20—N19	−176.90 (16)
C3—C21—C22—C23	142.1 (2)	C8—C3—C21—C22	165.31 (14)
C24—C21—C22—C23	−98.7 (2)	C3A—C3—C21—C22	42.6 (2)
C25—C21—C22—C23	17.8 (3)	C2—C3—C21—C22	−74.04 (19)
N14—C2—C3—C8	164.60 (14)	C8—C3—C21—C25	−70.57 (19)
N11—C2—C3—C8	45.63 (18)	C3A—C3—C21—C25	166.75 (14)
N1—C2—C3—C8	−73.52 (15)	C2—C3—C21—C25	50.1 (2)
N14—C2—C3—C3A	−85.22 (16)	C8—C3—C21—C24	46.82 (19)
N11—C2—C3—C3A	155.80 (14)	C3A—C3—C21—C24	−75.86 (19)
N1—C2—C3—C3A	36.66 (15)	C2—C3—C21—C24	167.47 (15)
N14—C2—C3—C21	41.0 (2)	C7—C7A—N1—O1	−35.1 (2)
N11—C2—C3—C21	−78.00 (18)	C3A—C7A—N1—O1	148.75 (13)
N1—C2—C3—C21	162.85 (14)	C7—C7A—N1—C2	−156.93 (16)
C8—C3—C3A—C4	−89.6 (2)	C3A—C7A—N1—C2	26.94 (16)
C2—C3—C3A—C4	160.80 (18)	N14—C2—N1—C7A	84.27 (15)
C21—C3—C3A—C4	35.6 (3)	N11—C2—N1—C7A	−162.41 (12)
C8—C3—C3A—C7A	87.46 (16)	C3—C2—N1—C7A	−39.85 (15)
C2—C3—C3A—C7A	−22.17 (16)	N14—C2—N1—O1	−38.44 (17)
C21—C3—C3A—C7A	−147.36 (14)	N11—C2—N1—O1	74.88 (16)
C7A—C3A—C4—C5	2.4 (2)	C3—C2—N1—O1	−162.57 (12)
C3—C3A—C4—C5	179.14 (17)	O10—C10—N11—C12	11.2 (3)
C3A—C4—C5—C6	−1.6 (3)	C9—C10—N11—C12	−165.97 (16)
C4—C5—C6—C7	−0.9 (3)	O10—C10—N11—C2	−174.82 (17)
C5—C6—C7—C7A	2.5 (3)	C9—C10—N11—C2	8.0 (2)
C6—C7—C7A—C3A	−1.7 (2)	C15—C12—N11—C10	−8.3 (3)
C6—C7—C7A—N1	−177.36 (15)	C13—C12—N11—C10	171.09 (15)
C4—C3A—C7A—C7	−0.7 (2)	C15—C12—N11—C2	177.29 (16)
C3—C3A—C7A—C7	−178.25 (15)	C13—C12—N11—C2	−3.37 (17)
C4—C3A—C7A—N1	175.62 (15)	N14—C2—N11—C10	−168.15 (14)
C3—C3A—C7A—N1	−1.89 (17)	N1—C2—N11—C10	72.10 (18)
C3A—C3—C8—C9	−126.85 (17)	C3—C2—N11—C10	−41.9 (2)
C2—C3—C8—C9	−21.7 (2)	N14—C2—N11—C12	6.74 (17)
C21—C3—C8—C9	104.56 (19)	N1—C2—N11—C12	−113.01 (14)
C3—C8—C9—O9	176.00 (16)	C3—C2—N11—C12	133.00 (14)
C3—C8—C9—C10	−9.9 (3)	O13—C13—N14—C2	−174.18 (15)
C8—C9—C10—O10	−158.30 (18)	C12—C13—N14—C2	6.41 (18)
O9—C9—C10—O10	16.7 (2)	N11—C2—N14—C13	−8.14 (18)
C8—C9—C10—N11	18.9 (2)	N1—C2—N14—C13	110.17 (15)
O9—C9—C10—N11	−166.11 (14)	C3—C2—N14—C13	−134.10 (15)
C15—C12—C13—O13	−1.8 (3)	N19—C18—N17—C16	1.5 (2)
N11—C12—C13—O13	178.90 (15)	C20—C16—N17—C18	−1.49 (18)
C15—C12—C13—N14	177.59 (17)	C15—C16—N17—C18	176.46 (16)
N11—C12—C13—N14	−1.72 (17)	N17—C18—N19—C20	−0.8 (2)
N11—C12—C15—C16	177.70 (16)	C16—C20—N19—C18	−0.2 (2)
C13—C12—C15—C16	−1.5 (3)		

### Hydrogen-bond geometry (Å, º)

**Table:**

*D*—H···*A*	*D*—H	H···*A*	*D*···*A*	*D*—H···*A*
N17—H17···O13	0.89 (2)	1.85 (2)	2.6562 (19)	151 (2)
N14—H14···N19^i^	0.83 (2)	1.97 (2)	2.798 (2)	175 (2)
C4—H4···O9^ii^	0.97 (2)	2.54 (2)	3.154 (2)	121.5 (16)
C20—H20···O13^iii^	1.00 (2)	2.54 (2)	3.353 (2)	137.9 (16)
C24—H24*C*···O13^iv^	0.98 (3)	2.47 (3)	3.382 (2)	154 (2)

Symmetry codes: (i) −*x*+1/2, −*y*, *z*−1/2; (ii) −*x*+1/2, −*y*+1, *z*−1/2; (iii) −*x*+1/2, −*y*, *z*+1/2; (iv) −*x*, *y*+1/2, −*z*+3/2.
